# HMQ‐T‐F2 exert antitumour effects by upregulation of Axin in human cervical HeLa cells

**DOI:** 10.1111/jcmm.13577

**Published:** 2018-03-07

**Authors:** Bingling Dai, Tianfeng Yang, Yujiao Ma, Nan Ma, Xianpeng Shi, Dongdong Zhang, Jie Zhang, Yanmin Zhang

**Affiliations:** ^1^ School of Pharmacy Health Science Center Xi'an Jiaotong University Xi'an China

**Keywords:** cervical HeLa cells, HMQ‐T‐F2, proliferation, Wnt/β‐catenin signal

## Abstract

Looking for novel, effective and less toxic therapies for cervical cancer is of significant importance. In this study, we reported that HMQ‐T‐F2(F2) significantly inhibited cell proliferation and transplantable tumour growth. Mechanistically, HMQ‐T‐F2 inhibited HeLa cell growth through repressing the expression and nuclear translocation of β‐catenin, enhancing Axin expression, as well as downregulating the Wnt downstream targeted proteins. Knock‐down of a checkpoint β‐catenin by siRNA significantly attenuated HeLa cell proliferation. Furthermore, XAV939, an inhibitor of β‐catenin, was used to treat HeLa cells and the results demonstrated that HMQ‐T‐F2 inhibited proliferation and migration via the inhibition of the Wnt/β‐catenin pathway.

## 1 INTRODUCTION

Cervical cancer is the fourth most common type of cancer and has the highest mortality rate among cancers in women.^1^ The conserved Wnt/β‐catenin signal transduction pathway is a critical pathway in several types of cancers, including cervical cancer,^2^ and controls many biological processes.^3^ Whether the Wnt/β‐catenin pathway is activated or not depends on the stability of β‐catenin in the cytoplasm. β‐catenin is regulated by a degradation complex that consists of the scaffolding protein Axin, which recruits essential elements during this process, such as the tumour suppressor adenomatous polyposis coli gene product (APC), casein kinase 1 (CK1) and glycogen synthase kinase 3β (GSK3β).

**Figure 1:**
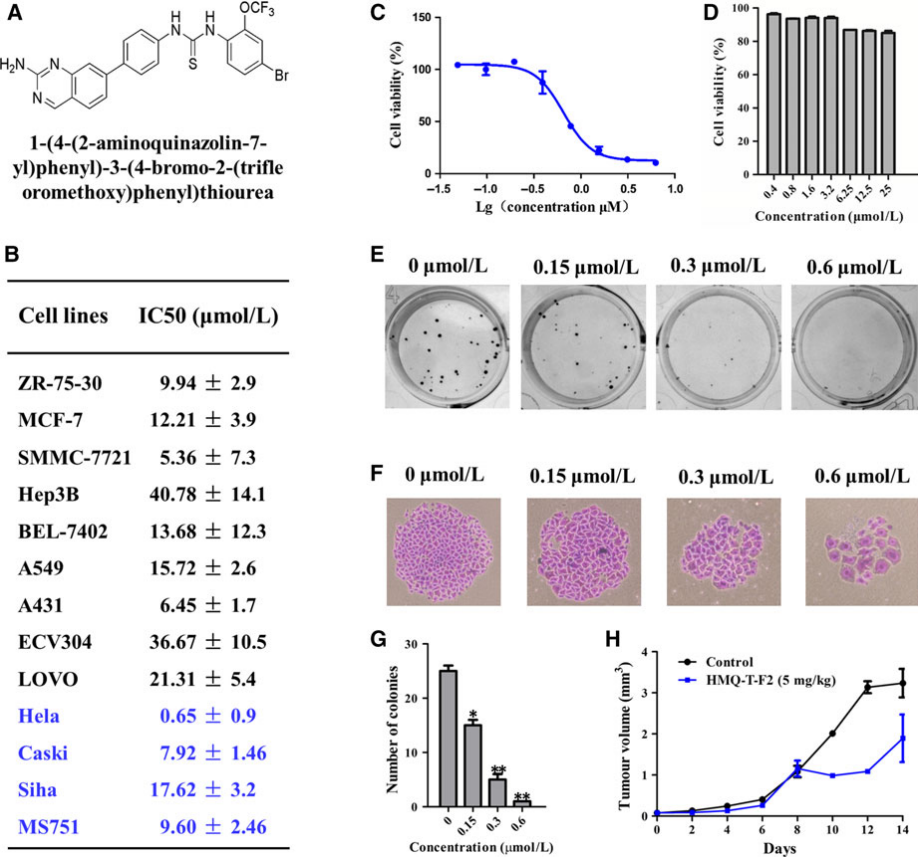
HMQ‐T‐F2 inhibited cell proliferation in vitro and in vivo. (A) Chemical structures of HMQ‐T‐F2. (B, C) Different human cancer cells (B), HeLa cells (C) and Human normal cervical epithelial cells (D) were treated with different concentrations of HMQ‐T‐F2 for 48 h. Cell viability was determined by the MTT assay and IC50 values were calculated. Data were presented as mean ± SEM. (E, F) Effect of HMQ‐T‐F2 on the colony formation of HeLa cells. The colony formation (E) and the individual colony (F) (200× magnification) were photographed. (G) Quantitation of the data represented in (E). (H) Effect of HMQ‐T‐F2 on the growth of HeLa xenografts. Mean tumour volume ± SEM at a given time‐point. Values are presented as means ± SEM. (n = 3). *P <* *.05, **P <* *.01 vs. the control group

Depending on the type and stage of cancer, treatments for cervical cancer include surgery, radiation therapy, chemotherapy and targeted therapy. Chemotherapy is often used to treat tumours that have spread or have reappeared after treatment.^4^ Therefore, there is an urgent need for more effective and less toxic therapies that could be represented by molecular target‐directed drugs. HMQ‐T‐F2(F2)(1‐(4‐(2‐aminoquina zolin‐7‐yl)phenyl)‐3‐(4‐bromo‐2‐(trifluoromethoxy)phenyl)thiourea) (Figure 1A) is a novel biphenyl urea derivatives designed using dissection strategies in our laboratory.^5^ In this study, we investigated the effect of HMQ‐T‐F2 on cervical tumour cell proliferation and explored in more depth HMQ‐T‐F2 as novel inhibitors of the Wnt/β‐catenin signal pathway.

## 2 MATERIALS AND METHODS

### 2.1 Chemicals and cell lines

HMQ‐T‐F2 (purity > 98%) were synthesized in the Research and Engineering Center for Natural Medicine, Xi'an Jiaotong University. ZR‐75‐30, MCF‐7, SMMC‐7721, Hep3B, Bel‐7402, A549, A431, ECV304, LOVO, and human cervical cancer cell line HeLa, Ca Ski, SiHa and MS751 were obtained from Shanghai Institute of Cell Biology in the Chinese Academy of Sciences. Human normal cervical epithelial cells were purchased from CHI Scientific, Inc (Jiangyin, China) and grown in DMEM/F12 medium with 10% (v/v) FBS and ECGS, Hydrocortisone, Heparin supplements. ZR‐75‐30, Hep3B, ECV304 and LOVO cell lines were cultured in DMEM medium with 10% (v/v) FBS; MCF‐7, SMMC‐7721, Bel‐7402, Ca Ski and HeLa cells were cultured in RPMI‐1640 medium with 10% (v/v) FBS; A431 and A549 cells were grown in F12 medium with 10% (v/v) FBS; SiHa and MS751 cells were cultured in MEM medium with 10% (v/v) FBS. All cell lines were incubated at 37°C in a 5% CO_2_ atmosphere with saturated humidity.

Four to six week old NOD/SCID nude male mice were purchased from Hunan SJA Laboratory Animal Co., Ltd. (Hunan, PR. China) and housed in the Experimental Animal Center of Xi'an Jiaotong University.

### 2.2 Cell proliferation assay

ZR‐75‐30, MCF‐7, SMMC‐7721, Hep3B, Bel‐7402, A549, A431, ECV304, LOVO, Ca Ski, SiHa, MS751, HeLa and human normal cervical epithelial cells were seeded and cultured with HMQ‐T‐F2 for 48 hours. Cells were cultured with MTT and analysed using a microplate reader (Bio‐Rad, Hercules, CA, USA).

### 2.3 Colony‐forming assay

HeLa cells were seeded and treated with HMQ‐T‐F2 for 48 hours. The colonies were stained with crystal violet and photographed.

### 2.4 In vivo assay

Mice were injected with 2 × 10^6^ HeLa cells. Mice were randomly assigned and respectively treated with vehicle (0.5% CMC‐Na), HMQ‐T‐F2 (5 mg/kg every day by intragastric administration. Mice weight and tumour volume were monitored every other day. After 14 days, mice were euthanized, and the tumours weights were recorded.

### 2.5 RNA extraction and real‐time quantitative RT‐PCR analysis

Total RNA was extracted from cells treated with HMQ‐T‐F2 and was reverse transcribed into cDNA. RT‐PCR was performed to evaluate the expression of the indicated genes on a Thermal Cycle Dice Real‐time PCR system (Bio‐Rad Laboratories, Inc., CA). The relative mRNA expression levels were calculated by the comparative 2^−ΔΔCt^ method and normalized against GAPDH expression.

### 2.6 Western blot analysis

After treatment, HeLa cells were collected and lysed in RIPA buffer. The samples were separated by SDS‐PAGE electrophoresis.

### 2.7 Immunofluorescence detection

After incubation with HMQ‐T‐F2 for 48 hours, HeLa cells were fixed and incubated with a primary antibody against β‐catenin (1:200) at 4°C overnight. Then, cells were incubated with an FITC‐labelled anti‐rabbit secondary antibody (1:50) for 1 hour. The nucleus was stained with DAPI. Fluorescent signals were detected using an inverted fluorescence microscope (DM505, Nikon Co., Ltd., Otawara, Tochigi, Japan).

### 2.8 siRNA transfection

Specific knockdown was achieved using a double‐stranded siRNAs against β‐catenin and nonspecific siRNA which were obtained from Shanghai GenePharma Co., Ltd (Shanghai, China). Transfected cells were subjected to RT‐PCR and western blotting. The transfected cells were used for cell proliferation assay.

### 2.9 Statistical analysis

One‐way analysis of variance (ANOVA) and further Tukey's multiple comparison test as well as independent‐samples t test were used. A *P* value <.05 was considered statistically significant. *P <* *.05, **P <* *.01, ***P <* *.001 compared to control group, ^#^ *P *<* *.05, ^###^ *P *<* *.001 compared to HMQ‐T‐F2‐treated groups, ^&&&^ *P *<* *.001 compared to XAV939‐treated groups. Data are expressed as means ± SEM.

## 3 RESULTS AND DISCUSSION

Chemotherapy plays a vital role in treating cervical cancer that has spread or has relapsed after treatment.^6^ Thus, searching for more effective and β‐catenin target‐directed drugs is an important issue in anti‐cervical cancer drug development. In this study, we found that our newly synthesized compound HMQ‐T‐F2 significantly reduced cell growth in a panel of cancer cell lines, including ZR‐75‐30, MCF‐7, SMMC‐7721, Hep3B, Bel‐7402, A549, A431, ECV304, LOVO Ca Ski, SiHa, MS751 and HeLa cells (Figure 1B). Interestingly, HMQ‐T‐F2 had a more potent inhibitory effect on HeLa cells (Figure 1C) and had no inhibition on human normal cervical epithelial cells (Figure 1D). Furthermore, our results showed that HMQ‐T‐F2 suppressed HeLa cell colony formation (Figure 1E‐G). At the same time, HMQ‐T‐F2 inhibited xenografts in nude mice in vivo (Figure 1H).

**Figure 2:**
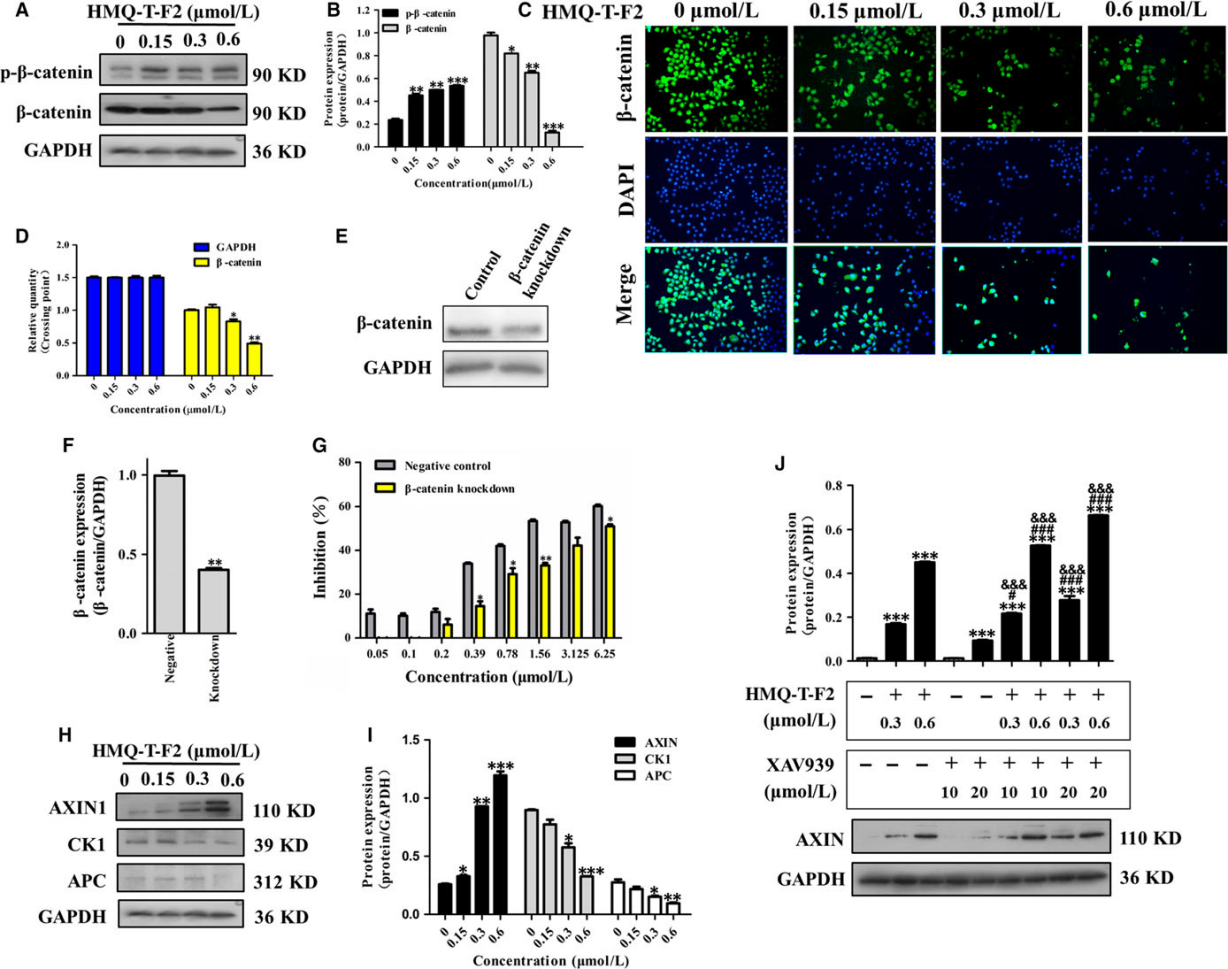
HMQ‐T‐F2 inhibited the expression and transactivation function of β‐catenin in HeLa cells. (A, B) The expression of phosphorylated β‐catenin (p‐β‐catenin) and β‐catenin in HeLa cells were detected by western blot. (B) Quantification of A. (C) HeLa cells were treated with different concentrations of HMQ‐T‐F2 and β‐catenin nuclear translocation was detected by immunofluorescence. FITC‐conjugated secondary antibody staining indicated the location of β‐catenin (green), DAPI staining indicated the location of the nucleus (blue), and the merged image indicates the nuclear location of β‐catenin protein (D) HMQ‐T‐F2 suppressed β‐catenin mRNA level in HeLa cells. (E, F) β‐catenin mRNA expression in HeLa cells transfections with 40 nM β‐catenin siRNA using Lipofectamine 2000 reagent and wild‐type HeLa cells were determined by western blot. (F) Quantitation data of (E). (G) Effect of HMQ‐T‐F2 on cell proliferation in wild‐type HeLa cells and β‐catenin knockdown HeLa cells. (H, I) Western blot analysis of cell cytoplasm protein included Axin, APC, CK1 and phosphorylation of GSK3β in Wnt signalling pathway of HeLa cells with quantification by relative densitometric values normalized to GAPDH. (**I**) Quantitation data of (H). (J) HMQ‐T‐F2 downregulated β‐catenin through stabilizing Axin. HeLa cells were treated with indicated concentrations of XAV939 for 24 h, followed by treatment of HMQ‐T‐F2 for 48 h. Data are expressed as mean ± SEM. *P <* *.05, **P <* *.01, ***P <* *.001 compared to control group, ^#^ *P *<* *.05, ^###^ *P *<* *.001 compared to HMQ‐T‐F2 treated groups, ^&&&^ *P *<* *.001 compared to XAV939‐treated groups

β‐catenin, a central mediator in the Wnt/β‐catenin signalling pathway, is expressed in many solid tumours.^7^, ^8^ Nuclear translocation of β‐catenin is considered a marker of pathway activation.^9^ Previous study has shown strong β‐catenin staining in cervical cancer tissues by immunohistochemistry.^2^ Here we found that HMQ‐T‐F2 not only reduced the protein level of β‐catenin but also significantly suppressed the nuclear translocation of β‐catenin (Figure. 2A‐C), which resulted in decreasing mRNA levels (Figure 2D). Meanwhile, the inhibitory effect on HeLa cells (wild type) was much greater than that of β‐catenin knockdown HeLa cells (Figure 2E‐G). It indicated that β‐catenin is a key target for HMQ‐T‐F2.

The stability of the Wnt pathway transcription factor β‐catenin is tightly regulated by the multi‐subunit destruction complex. The β‐catenin destruction complex, which consists of Axin, APC, CK1 promotes proteasome‐mediated proteolysis mediated proteolysis of phosphorylatedβ‐catenin.^10^ Axin is a negative regulator of the Wnt signalling pathway, which promotes the phosphorylation and degradation of β‐catenin. Our results showed that HMQ‐T‐F2 significantly increased the expression of Axin, decreased the expression of APC, CK1 (Figure 2H,I). XAV939, a small molecule inhibitor of Wnt signalling pathway, acts through stabilization of the Axin protein and stimulation of β‐catenin degradation.^11^ When pre‐treated with XAV939 before exposure to HMQ‐T‐F2, we found that HMQ‐T‐F2 could enhance XAV939‐induced activity and further indicated that HMQ‐T‐F2 mediated β‐catenin degradation by stabilizing Axin.

In summary, this study demonstrated that HMQ‐T‐F2 inhibited cervical HeLa cell proliferation in vitro and in vivo. The mechanism underlying these effects involves β‐catenin degradation and maintenance of Axin stability by HMQ‐T‐F2 and therefore, the inhibition of the Wnt/β‐catenin signalling pathway. This study suggests that HMQ‐T‐F2 potentially have a therapeutic value for cervical cancer treatment.

## CONFLICT OF INTEREST

The authors declare that they have no conflict of interests to disclose.

## AUTHOR CONTRIBUTIONS

BLD, TFY and YJM performed the main experiments and summarized the results. NM and XPS assisted in performing the experiments. DDZ assisted in interpreting the data. JZ supplied the compound. BLD wrote the manuscript. BLD and YMZ provided the concept, funding, supervision and assisted in writing the manuscript. All authors read and approved the final manuscript.
